# Sexually dimorphic microglia and ischemic stroke

**DOI:** 10.1111/cns.13267

**Published:** 2019-11-20

**Authors:** Nadine Kerr, Dalton W. Dietrich, Helen M. Bramlett, Ami P. Raval

**Affiliations:** ^1^ Department of Neurological Surgery Leonard M. Miller School of Medicine University of Miami Miami FL USA; ^2^ Bruce W. Carter Department of Veterans Affairs Medical Center Miami FL USA; ^3^ Peritz Scheinberg Cerebral Vascular Disease Research Laboratory Department of Neurology Leonard M. Miller School of Medicine University of Miami Miami FL USA

**Keywords:** estrogen receptors, inflammasome, inflammation, reproductive senescent female

## Abstract

Ischemic stroke kills more women compared with men thus emphasizing a significant sexual dimorphism in ischemic pathophysiological outcomes. However, the mechanisms behind this sexual dimorphism are yet to be fully understood. It is well established that cerebral ischemia activates a variety of inflammatory cascades and that microglia are the primary immune cells of the brain. After ischemic injury, microglia are activated and play a crucial role in progression and resolution of the neuroinflammatory response. In recent years, research has focused on the role that microglia play in this sexual dimorphism that exists in the response to central nervous system (CNS) injury. Evidence suggests that the molecular mechanisms leading to microglial activation and polarization of phenotypes may be influenced by sex, therefore causing a difference in the pro/anti‐inflammatory responses after CNS injury. Here, we review advances highlighting that sex differences in microglia are an important factor in the inflammatory responses that are seen after ischemic injury. We discuss the main differences between microglia in the healthy and diseased developing, adult, and aging brain. We also focus on the dimorphism that exists between males and females in microglial‐induced inflammation and energy metabolism after CNS injury. Finally, we describe how all of the current research and literature regarding sex differences in microglia contribute to the differences in poststroke responses between males and females.

## 1 INTRODUCTION

Ischemic stroke is the third leading cause of death in developing countries and is a major cause of disability globally. Close to 15 million people are affected by stroke worldwide every year, of which approximately 5 million die and 5 million suffer from long‐term disability.^1^ It is known that gender plays an important role in the pathogenesis and outcome of stroke. The incidence of stroke in women is a growing public health issue. Statistics show that stroke affects women more than men, is the second cause of death in women over 60, and represents 60% of all stroke‐related deaths.^2^ Elderly stroke patients may be more likely to suffer from severe neurological deficits and complications. Of these patients, women more frequently need assistance with daily living activities and are more likely to live in nursing homes after stroke.^2^ In addition, females are more likely to suffer from recurrent and more severe strokes.^2^

Currently, tissue plasminogen activator (tPA) is the only FDA‐approved pharmacological intervention used as a treatment option for stroke. However owing to the narrow time window for treatment poststroke, less than 10% of patients qualify for tPA administration.^3^ Advances in intravascular procedures such as thrombectomy have increased the number of patients suitable for clot removal strategies while increasing the therapeutic window for intervention.^4^ The sex disparities in stroke pathogenesis may also play a role in finding the best treatment options. Recently, research has focused on developing other forms of treatment for stroke that target gender differences in neuronal and glial cell death and the inflammatory response.^5^ Increasing evidence shows that the inflammatory response is key to understanding the pathobiology of stroke at any stage after the ischemic event.^6^ Microglia play a role in regulating the propagation and resolution of the inflammatory response after CNS injury. It is clear that microglial activation plays an important role in the inflammatory response after stroke in both males and females.^7^ However, more recent research suggests the possibility of a sexual dimorphism in the neuroinflammatory response after stroke.

Microglia are known as the resident brain macrophages and comprise about 5%‐12% of total brain cells, depending on the brain region.^8^ Under normal conditions, microglia exist in a resting state under what is known as a ramified morphology.^9^ They are involved in regulating neuronal excitability, synaptic activity, connectivity, neurogenesis, and clearance of apoptotic cells in the healthy adult brain.^10^ In this review, we will focus on the role of microglia as a modulator of the differences in the inflammatory response and outcomes poststroke. We will also focus on the sex differences in microglia between the developing and aging brain and how this may contribute to the clinical evidence indicating that aged females suffer more detrimental outcomes poststroke. Finally, we will discuss the sex differences between microglia‐regulated inflammatory signaling as well as the role of sex hormones on microglial activation and how this may contribute to the sexual dimorphism that is seen in stroke pathobiology and outcome.

## 2 MICROGLIAL SEX DIFFERENCES IN DEVELOPING BRAIN

Microglia arise early during embryonic development. In particular, lineage studies have established that microglia are derived from primitive myeloid progenitors that arise before embryonic day 8.^11^ During the early embryonic period, microglia are mainly localized to proliferative zones of the brain^12^ and play an important role in stem cell pools and neurogenesis via secretion of trophic factors and phagocytosis.^11^ In vitro studies have even demonstrated that without microglia present, inducible neurogenesis does not occur.^13^ Toward the end of perinatal development, microglia play a role in phagocytosis of both apoptotic and healthy nonapoptotic neural precursor cells, and therefore, they restrain overpopulation of these cells in developing brain.^11^ After the prenatal period, microglia continue to develop through the early postnatal period. Rodent studies have determined that microglia proliferate locally until the second postnatal week, and then, numbers start decreasing to adult levels.^14^, ^15^ Another very important role for microglia during postnatal development is their involvement in synapse formation throughout the brain. More recently, microglia have been indicated in synapse remodeling in the cortex and hippocampus^16^ as well as the retinogeniculate circuit and thalamus.^17^ Following their initial colonization, microglia proliferate rapidly during perinatal development and continue to develop during adulthood.^18^

**Figure 1:**
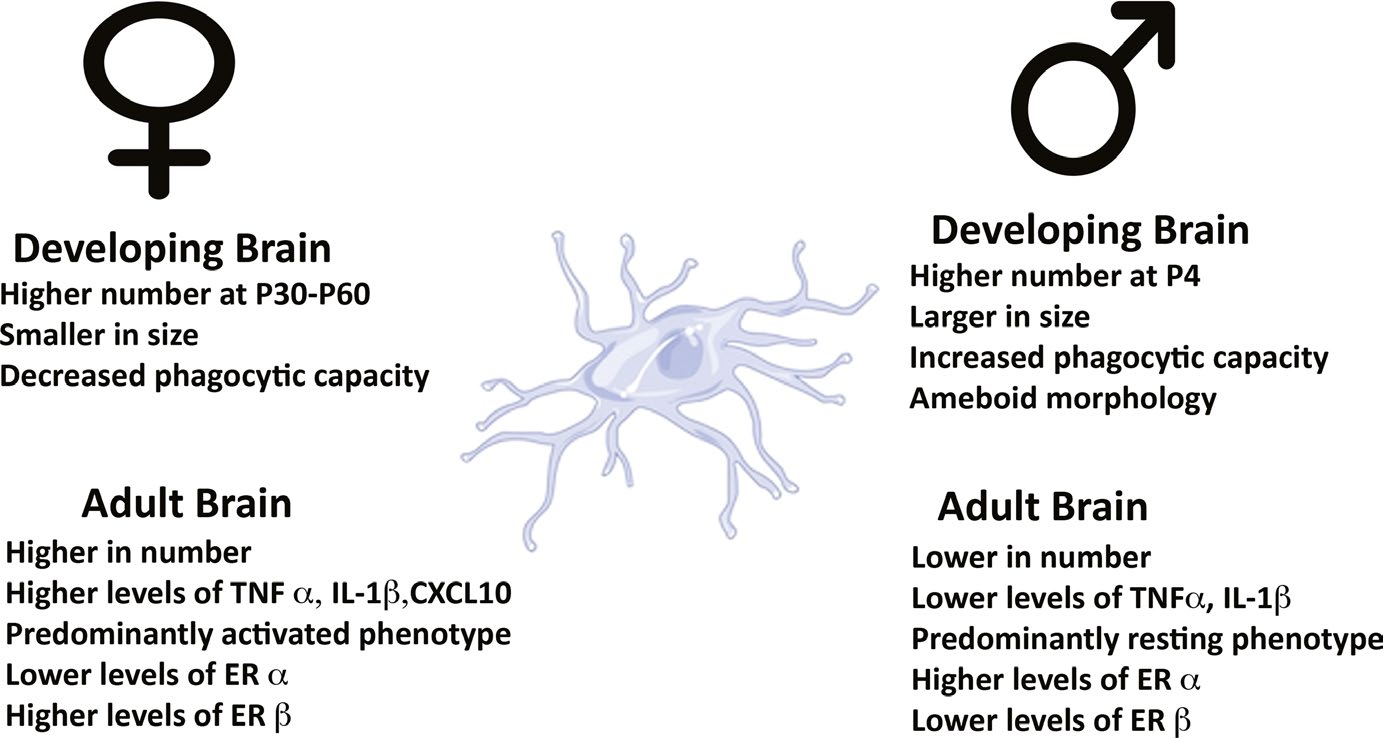
Sexual differentiation of developing and adult microglia. Schematic highlighting the main differences between males and females in developing and aged microglia

There is a known sex difference in microglial development, as males have more microglia than females in the developing neonatal period.^10^ It was recently shown that males have significantly higher numbers of microglia early in postnatal development (postnatal day 4), while females have more activated microglia later in development as juveniles and adults (postnatal days 30‐60) (Figure 1).^19^ Male rodents have a significantly higher number of microglia at postnatal day 4 in the parietal cortex, CA1, CA3, dentate gyrus, and amygdala, while female rodents show a higher number of microglia in these regions later in adulthood (Figure 1).^19^ All of these brain areas are involved with learning and memory and cognition. Rodent studies have shown that bacterial infection in male rat pups on postnatal day 4 causes significant long‐term changes in brain function, while females do not show this same response at postnatal day 4.^20^ The pattern shifts at postnatal days 30‐60, also associate with sex divergences in their gene expression profile.^21^ Evidence supporting a significant role of either sex chromosome or sex hormones plays a role in the observed sex differences in perinatal microglial development remains elusive. In general, sex hormones are known to reduce neuroinflammation, and therefore, a developmental sex difference in microglial proliferation might in part contribute to changing levels of androgens and estrogens.^22^ On the other hand, sex differences in microglial number might be a result of sex differences in chemotactic signals that differentially recruit the cells in males and females.^19^ Irrespective of the mechanism by which sex differences in microglial development is governed, microglia are important for the survival of new neurons and synaptic connections,^23^, ^24^ and these processes are sexually dimorphic within the developing and adult brain.^19^ This sexual dimorphism in microglia development in different brain regions may contribute to differences in male and female responses to neurological disorders such as ischemic stroke.

Here, it is important to note that studies from various laboratories have used microglial cultures from postnatal day 1 pups to mimicking the condition of ischemia by oxygen‐glucose deprivation (OGD) and tested cell‐protective properties of different drugs. Importantly, many of these studies tested mixed cultures having derived cells from both male and female pups. Thus, given the different developmental properties, their response to OGD or drugs may be different from what would have been in uninjured intact brains. Therefore, the results of such studies should be interpreted with caution, and future more rigorous studies using sex‐specific microglial cultures may add more information.

## 3 MICROGLIAL SEX DIFFERENCES IN HOMEOSTATIC ADULT BRAIN

Apart from neuroinflammation, microglia also carry out multiple other functions that also demonstrated aspects of sex difference and are described subsequently. Current research has found that microglia also play a novel role in adult neural plasticity and the regulation of long‐term potentiation (LTP) and long‐term depression (LTD).^10^ Studies have reported that increases in tumor necrosis factor alpha (TNFα) and interleukin‐1 beta (IL‐1β) lead to deficits in hippocampal LTP.^25^, ^26^ This can lead to neuroprotection as microglia can regulate neuronal damage after excessive synaptic activity by releasing repair factors in response to prolonged release of excitatory neurotransmitters.^27^ The early postnatal phase is a crucial window for synaptic formation, which explains why there is a sexual dimorphism in synaptic formation between males and females. This is consistent with data from rodent studies, which demonstrated that males contain a higher number of microglia in brain regions associated with cognition and learning and memory.^19^ Little is understood about the sexual differences in this aspect of microglial function. However, it is apparent that slight sex differences in cell size and phagocytic capacity to exist in the hippocampus of rodents at all stages of development.^17^

In addition to their role in synaptic development, microglia also play a role in adult neurogenesis and apoptosis. It has been determined that microglia can regulate neurogenesis and apoptosis under both normal and pathological conditions.^10^ Ramified microglia play a critical role in phagocytosis of apoptotic cells during the first few days of their life.^28^ Under this type of basal condition, microglia phagocytose newly born cells that are undergoing apoptosis in the subgranular zone of the hippocampus and the subventricular zone (SVZ) of the lateral ventricle.^29^ The clearance of cells is not the only way that microglia can promote neurogenesis. It is evident that microglia can also promote neurogenesis through the secretion of factors that enhance proliferation, differentiation into neurons or glia, and survival of newborn cells.^28^ A study demonstrated an instructive role for microglial cells in contributing to postnatal neurogenesis in the largest neurogenic niche of the mammalian brain. In this study, authors demonstrated that neurogenesis in highly expanded neural stem cells (NSCs) can be rescued by coculture with microglial cells or microglia‐conditioned medium, indicating that microglia provide secreted factor(s) essential for neurogenesis, but not NSC maintenance, self‐renewal, or propagation.^13^ It is possible that various growth factors may be responsible for the effect that microglia have on neurogenesis. In particular, growth factors, such as brain‐derived neurotrophic factor (BDNF) and insulin‐like growth factor 1 (IGF 1), are the key players in promoting neurogenesis.^30^ The role of microglia‐mediated IGF‐1 signaling has been implicated in stroke neuroprotection.^31^ In a rat model of stroke, microglia expressing IGF‐1 were seen in the SVZ of the ischemic hemisphere as early as 2 weeks after stroke and persisted up to 16 weeks poststroke.^32^ This neuroprotective mechanism may in part be due to the cooperative effect of estrogen and IGF‐1 signaling.^31^ Less is known about the role of microglial‐mediated IGF‐1 signaling and estrogen after stroke; however, a recent article suggests the possibility that estrogen and IGF‐1 act together on microglia to mediate an increase in IGF‐1 synthesis.^31^ It is also apparent that IGF‐1/IGF‐binding protein 3 declines faster in males than females,^33^ which may also contribute to the differences seen in microglial‐mediated IGF‐1 signaling between aged males and females. This notion is also supported by a study, in which IGF‐1 improves stroke recovery aged female rats.^34^

Aside from neurogenesis, microglia support cell genesis and regulate stem cell pools. For example, microglia can promote astrogliogenesis from stem cells in culture through the release of the anti‐inflammatory cytokine IL‐6.^35^ In addition, pharmacological suppression of the activation of microglia in vitro inhibited both neurogenesis and oligodendrogenesis in the SVZ.^36^ While the exact difference between microglial‐influenced neurogenesis between males and females is not fully understood, there are several factors, which suggest that a sexual dimorphism exits. Studies have determined that males have more microglia in ameboid morphology in the developing brain and that female microglia tend to reach an adult phenotype earlier than males (Figure 1).^37^ Supporting this view further, research has demonstrated that females engulf neural progenitor cells and healthy cells at a higher rate than males.^38^ This phenomenon may contribute to sex differences in neurogenesis.

In order for microglia to perform their many functions in the brain, a large amount of energy is required. Sex differences in microglial activation have been implicated in the modulation of energy homeostasis. This was shown in the hypothalamic microglial activated CX3CL1‐CXCLR1 pathway in a mouse model of obesity.^39^ Dorfman et al found that increased CXCLR1, which is associated with energy expenditure, allowed for females to be more resistant to obesity.^39^ This therefore supports the idea that differences in microglial function exist between males and females. Many brain disorders and injuries involve drastic changes in energy metabolism although inflammation is sustained. Furthermore, in order for microglia to survive, a sufficient energy supply is needed.^40^ Microglia require both glycolytic and oxidative energy metabolism, and microglia express a set of genes that are required for both types of metabolism.^41^ The essential fuel for microglia is glucose, which they are able to take up by various glucose transporters (GLUTs).^42^ Microglial function, in conjunction with glucose availability and glycolytic rate, is known to influence pro‐inflammatory gene expression at both the transcriptional and translational levels.^43^ All of these forms of energy consumption by microglia are important in maintaining brain homeostasis and are crucial for progression and repair of CNS injury and neurodegeneration.

## 4 SEX DIFFERENCES IN MICROGLIA‐INDUCED INFLAMMATION

It has long been understood that the brain is immune‐privileged in comparison with the rest of the body. Microglia contribute to this by responding to infectious agents or injury to the brain. Upon insult or infection, microglia take on an ameboid form, which is highly motile and allow microglia to swim to a site of injury very quickly.^10^ Microglia then release pro‐inflammatory cytokines in order to recruit additional immune cells to the site of infection or injury.^44^ A well‐known characteristic of activated microglia is their expression of two phenotypes: M1 and M2. M1 microglia are involved with a pro‐inflammatory response, while M2 microglia are immunosuppressive.^45^ In the “classical activation” state, pro‐inflammatory cytokines, including IL‐1β, TNFα, and IL‐6, are released by M1.^45^ M2 microglia are involved in the state of “deactivation” and are involved in the clearance of cell debris and misfolded proteins, promote extracellular matrix and tissue repair, and support neuronal survival through the release of neurotrophic factors.^45^ The role of both M1 and M2 microglial phenotypes is an important players in the inflammatory response poststroke.^7^ In the acute phase after ischemic injury, pro‐inflammatory proteins are released from the injured brain and endogenous microglia are then recruited and activated to polarize to the M2 phenotype.^46^ In vitro studies found that when M1 microglia were added to cell culture, there was increased OGD‐induced neuronal death; however, when M2 microglia were added, neurons were protected against OGD.^47^ This suggests that the regulation of both M1 and M2 phenotypes has an implication in stroke pathogenesis.

Depending on the microglial phenotype, they can either release cytotoxic or neuroprotective effects. Sex differences in the expression of cytokines and chemokines in other brain regions are also seen. For example CCL4, CCL20, and CD206 vary from male to female depending on the developmental age in the hippocampus, amygdala, and cortex.^19^ Genes for these inflammatory proteins, as well as IL‐1β, TNFα, and CXCL10, are more prominent in adult female mice, which are consistent with the understanding that females have more microglia than men in later stages of development.^48^ Furthermore, one study demonstrated that upon lipopolysaccharide (LPS) stimulation, baseline morphological characteristics of male microglia were significantly reduced and those of females did not have significant changes.^49^ Knowledge of these age and sex‐dependent differences in activated microglia in males and females is required to form a comprehensive basis of ischemic brain pathology.

It has been demonstrated that microglia polarize under optimal condition and is highly temperature dependent after the brain injury.^50^ The effects of targeted temperature management appear to be sex‐dependent and have potential to be one of the most attractive therapies for ischemic stroke.^51^ Pioneering studies from our laboratory have demonstrated that lowering of brain temperature by only a few degrees can ameliorate ischemic neuronal death.^52^ Therapeutic hypothermia has been widely regarded to be one of the most reliable neuroprotective therapies for several cerebral disorders and injuries, including stroke, traumatic brain and spinal cord injury, global ischemia after cardiac arrest, and hypoxic‐ischemic encephalopathy.^53^, ^54^, ^55^, ^56^, ^57^ The ischemic injury stimulates innate immune responses leading to activation of microglia and circulating leukocytes, and these immune cells can then release various molecules, including ROS, proteases, and pro‐inflammatory cytokines. These molecules can activate more inflammatory cells, leading to a vicious cycle of death and inflammatory activation.^50^, ^55^ In recent years, the importance of the innate immune response in brain injury has been emphasized and targeted for therapeutic interventions.^50^ A key component of the innate immune response is the inflammasome,^58^, ^59^ which is a multiprotein complex responsible for the activation of caspase‐1 and processing of inflammatory cytokines IL‐1β and IL‐18.^60^, ^61^ The nod‐like receptor 3 (NLRP3) inflammasome has been studied in CNS injury, including traumatic brain injury and stroke, and has recently been indicated in neuronal and glial damage poststroke.^62^ Microglia have shown to upregulate NLRP‐3 inflammasome gene expression 24 hours after inflammation‐sensitized hypoxic‐ischemic brain injury.^63^ The inflammasome activation and the subsequent release of IL‐1β induce pyroptosis‐cell death pathway.^64^ In a recent study, using a rat model of penetrating brain injury we confirm that microglial activation plays a role in pyroptotic cell death.^64^ One of the potential therapeutic approaches based on aforementioned findings could be to use M2‐like microglial cell therapy for stroke. The M2‐like microglia and monocytes/monocytes secrete protective remodeling factors, thus prompting neuronal network recovery via tissue/vascular remodeling; however, future translational studies are required.^65^

A more recent area of neuroinflammatory research has been the role of extracellular vesicles, which are small nano‐to‐micrometer vesicles involved in cell‐to‐cell communication. It is understood that extracellular vesicles (EVs) play a critical role in coordinating communication between microglia, neurons, and astrocytes.^66^ EVs can be derived from various types of cells in the body, including microglia. It is known that EVs can carry both pro‐inflammatory as well as neuroprotective factors. Recent studies from our group have demonstrated that EV‐mediated inflammasome signaling plays a role in the pathogenesis of TBI‐ischemic damage and TBI‐related systemic complications.^67^ We have also shown that inflammasome proteins are increased in serum‐derived EVs from stroke patients compared with control patients in the acute phase poststroke and that they can be used as a potential biomarker for stroke diagnosis.^68^ In the case of microglial‐derived EVs, it has been determined that stimulation with LPS significantly increased microvesicle release by microglia, whose content is enriched with IL‐1β and can drive a pro‐inflammatory response.^69^ Microglial‐derived EVs have also been shown to regulate synaptic transmission by promoting neural production of ceramide and sphingosine.^70^ It is evident that EVs can be derived from microglia from both M1 and M2 phenotypes and that M2‐derived EVs show a protective effect after ischemic attack through the release of trophic factors.^71^ There is little research regarding sex differences in EVs, and therefore, further research is needed to examine sexual dimorphism in microglial‐derived EVs.

## 5 MICROGLIAL SEX DIFFERENCES DURING AGING

Changes in microglial function due to aging are very important contributing factors to neurodegeneration and aging‐related CNS disorders. Microglia shows age‐dependent cellular dystrophy and become senescent.^72^ This senescence in microglia leads to functional changes that may be responsible for an age‐dependent increase of microglia‐mediated neuroinflammatory response, which can also damage aged neurons.^73^ Various studies have investigated age‐dependent changes in the number of microglia in different brain regions across different species. One study reported that there were no changes in Iba1, a microglial marker, in the hippocampus of aged rats.^74^ However, another study showed a decrease in Iba1 in the nigrostriatal system and cerebral cortex in rodents.^75^ While it is still unclear on whether or not changes in microglial morphology are signs of neurodegeneration, it was recently reported that changes in microglial dystrophy reflect age‐related cytoskeletal alterations.^76^

Age‐related morphological changes in microglia can also affect inflammatory responses after CNS injury and disease. Microglia in mutant (Ercc1) mice that display features of accelerated aging showed hallmark features of priming and increased responses to LPS stimulation along with increased cytokine expression and cell death.^77^ The pro‐inflammatory cytokines, IL‐1β, TNFα, and IL‐6, were all increased after LPS challenge in aged microglia.^78^ Other signs of increased age‐dependent microglia are the expression of immune cell markers such as MHCII, macrophage marker CD68, and Toll‐like receptors (TLRs), which all play a role in the inflammatory response after CNS injury.^73^ TLRs have been implicated in the role of both the detrimental and protective roles after ischemia and are involved in the activation of microglia poststroke.^79^ For example, microglial activation poststroke has been observed as a result of TLR2 and TLR4 activation.^80^ In addition, suppression of TLR activating pathways, through Tyro3, Axel, and Mer (TAM receptors), has shown promise as therapeutic targets for reducing microglial‐regulated inflammation post‐MCAO in a rodent model.^81^ Microglia also play a role in ischemic preconditioning (IPC), which is protective against prolonged ischemic events. TLRs are some of the key signaling pathways in IPC reprogramming of the CNS.^82^ A study demonstrated that pretreatment with unmethylated CpG oligodeoxynucleotides (ODNs)—a ligand for Toll‐like receptor 9 (TLR9)—induces protection against ischemic brain damage in mice and nonhuman primates.^83^, ^84^ However, it has been determined that CpG‐ODN induced cerebral ischemia tolerance only in male mice, not in females, which indicates a sex difference.^83^ Studies implicate TLR4 activation leads to a number of downstream events that are necessary for the establishment of IPC and the microglial response to damage.^82^ Supporting this idea, studies have shown that aged mice have increased microglial activity after injection of IL‐1β and IL‐12 into the hippocampus.^85^ This phenomenon is known as microglia priming and can make the microglia susceptible to secondary inflammatory stimulus leading to an exaggerated inflammatory response after CNS injury.^73^ These changes in microglial‐related inflammatory responses after aging occur in both males and females. Imaging studies demonstrated that TLR2 is increased in both male and female rodents after ischemic injury and that these levels are significantly higher when compared to younger rodents.^6^ However, postmenopausal females are known to be susceptible to stroke, due to the loss of the neuroprotective effect of estrogen after menopause.^6^ Estrogen receptor α (ERα) knockout mice showed dysfunctional microglial activation and an increased pro‐inflammatory response after ischemic injury.^86^ This suggests that differences in aged‐microglial responses between males and females do exist and that other factors, such as hormones, may contribute to this dimorphism.

## 6 EFFECTS OF SEX HORMONES ON MICROGLIA

Differences between sex hormones in males and females are a contributing factor to several developmental differences between the two sexes. The exact role of sex hormones and the maturation of microglia are not yet fully understood. Both male and female sex hormones have been implicated in differences seen in the microglial response to neurological insult. Testosterone, a male sex hormone, has been indicated in the microglial response to CNS injury. It has been determined that depletion of testosterone in male mice increased BBB permeability, which was accompanied by the increase in activation of astrocytes and microglia.^87^ Activation of microglia in these mice was accompanied by an increase in pro‐inflammatory proteins such as COX‐2, iNOS, IL‐1β, and TNFα.^87^ Therefore, it is possible that the absence of physiological levels of testosterone, microglia may increase the pro‐inflammatory response in the brain leading to a loss of BBB integrity after CNS injuries, such as stroke.

It is known that microglial expression of sex hormone receptors is relative to the stage of brain maturation.^88^ In addition, higher ERα levels have been found in microglia from P3 mice and these levels continue to increase with age, even though no sex differences were seen at any age.^89^ Lenz et al conducted a key experiment in understanding the role of microglia in sexual differentiation of the brain where they discovered that perinatal treatments with minocycline (an inhibitor of microglial activity) prevented masculinization of the brain that normally occurs with estradiol.^37^ This was determined by the prevention of sex differences in microglia, estradiol‐induced masculinization of dendritic spine density, and adult copulatory behavior.^37^ Supporting this, more recent studies have implied that microglia are modulated by male and female sex hormones. Villa et al demonstrated that when microglia isolated from adult rodent brain were placed in culture or put into the brains of the opposite sex; they maintain the same sex‐specific features.^90^ These previous studies suggest that microglia interact with the endocrine system during development and that they play an important role in sexual differentiation in the central nervous system. Apart from systemic hormonal influence, the CNS is a highly steroidogenic environment synthesizing steroids de novo, as well as metabolizing steroids derived from the circulation. The synthesis of neurosteroids is regulated by neuroinflammation, and on the contrary, several steroids, for example 17β‐estradiol, dehydroepiandrosterone (DHEA), and allopregnanolone, regulate neuroinflammatory responses mediated by microglia.^91^

Estrogen, a female sex hormone, is known to have neuroprotective effects. Recent literature provides evidence that estrogens and estrogen receptors are involved with microglial action.^88^ Studies performed in microglial cell lines showed that mRNA of both types of estrogen receptors, ERβ and ERα, were expressed and that content changed significantly with passages in culture.^92^ Furthermore, the anti‐inflammatory characteristics of ERs and estrogens inhibited pro‐inflammatory cytokine expression in microglia that were stimulated with LPS.^93^ In vivo studies focusing on the role of estrogen and ERs with microglia have also provided strong evidence that they play an important role in microglial‐inflammatory responses. For example, one study showed that ovariectomy (ovx) in rodents is associated with more microglia containing pro‐inflammatory morphology and that delivery of estradiol before ovx blocked microglial activation.^94^ This suggests that the microglial‐mediated inflammatory response after ovx was due to lack of this hormone. Furthermore, ovx mice express a significantly higher amount of pro‐inflammatory mediators than intact mice of the same age, which indicates that low levels of estrogen increase the susceptibility of microglial activation and neuroinflammation.^88^ Collectively, these data support the hypothesis that estrogen and microglial ERs activation have anti‐inflammatory effects that can enhance the neuroprotective properties of microglial action.

The depletion of endogenous estrogen at reproductively senescence increases innate immune—inflammasome proteins in the brain of females but not in age‐matched male rats.^95^ Recently published studies from our laboratory demonstrated that in reproductively senescent females, EVs originating from the reproductive organs carry inflammasome proteins and spread to the brain, thus producing an exacerbated innate immune response in the brain which may be responsible for the increased severity of stroke.^95^ It is also demonstrated that ER‐β regulates inflammasome activation in the brain of female rats. Silencing of ER‐β attenuated 17β‐estradiol‐mediated decrease in caspase 1, ASC, and IL‐1β. On the contrary, periodic ER‐β agonist treatment reduces inflammasome activation and ischemic damage in reproductively senescent female rats.^96^ The aforementioned studies did not demonstrate a direct role of microglial ER‐β in inflammasome activation in the female brain. However, this possibility cannot be ruled out, as anti‐inflammatory activities of 17β‐estradiol are mediated by its receptors ER‐α and ER‐β.^97^, ^98^

## 7 MICROGLIAL SEX DIFFERENCES AND STROKE

Microglia play a critical role in the progression and resolution of neuroinflammation after ischemic brain injury. This is supported by a recent study, which determined that when microglia were depleted in mice using a colony‐stimulating factor receptor (CSF1R), stroke outcomes were much worse compared with normal mice.^99^ Microglia undergo a rapid morphological change after injury, characterized by a transformation from a ramified to ameboid form, which is highly motile and allows microglia to reach the site of injury within minutes after insult.^10^ Once microglia arrive at the site of injury, they engage in a robust inflammatory response and release pro‐inflammatory cytokines that will then recruit additional immune cells.^100^ After ischemic injury, microglia produce pro‐inflammatory cytokines and neurotoxic molecules including IL‐1β, TNFα, and ROS.^101^ Microglia depletion in mice was associated with increased leukocyte infiltration and higher levels of pro‐inflammatory cytokines.^99^ Yet on the other hand, microglia can also be involved with neuroinflammatory repair after CNS injury. Anti‐inflammatory mediators such as IL‐4 and IL‐10 are produced to allow repair after ischemic injury.^102^ After propagation of the inflammatory response, they will phagocytose dead and dying cells in order to limit the spread of damage.^88^ In addition, microglia contribute to resolution of neuroinflammation by engaging in anti‐inflammatory signaling and growth factor secretion.^100^ Furthermore, both the M1 and M2 microglial phenotypes play a role in the inflammatory response after CNS injury, even though the extent to which each phenotype is involved is not yet fully understood.^103^

Dimorphism in neuroinflammatory responses between males and females is known to exist after ischemic injury. This is evident through differences in the molecular mechanisms of neuroinflammation after stroke between males and females. For example, there is a known sexual dimorphism in stroke and the mechanisms behind this phenomenon are not yet fully understood. Females have a lower incidence of stroke (age‐dependence), even though they may have worse outcome and show more functional decline when compared to males.^1^ Furthermore, when microglia from female mice were transplanted into male mice, the progression of injury poststroke was reduced.^90^

**Figure 2:**
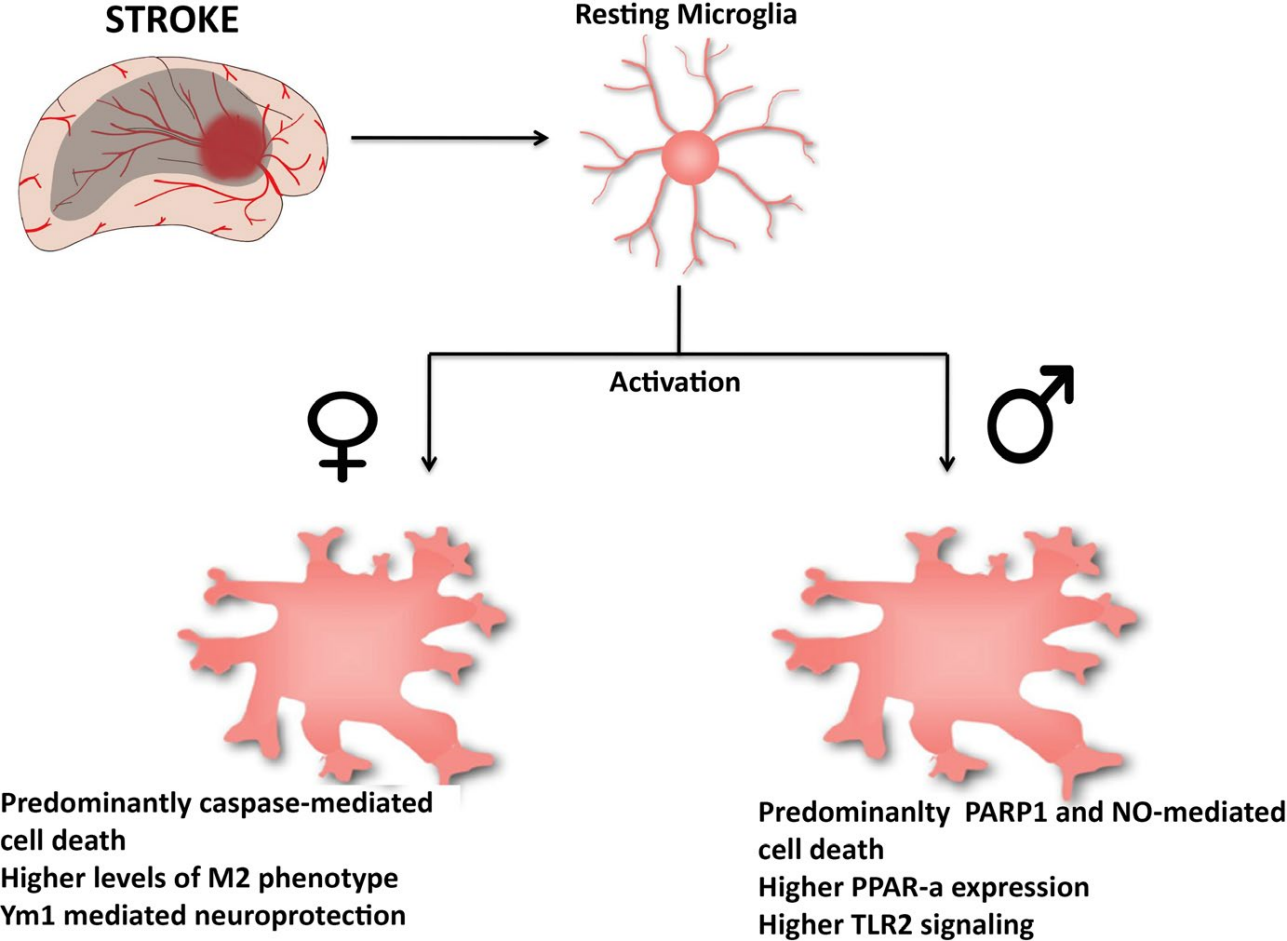
Sexual differences in activated microglia poststroke. Schematic representations of the main characteristics seen in activated microglia after stroke in males and females

Microglia have been implicated to play a role in the sexual dimorphism that exists in stroke in preclinical studies. After a rodent MCAO model of acute stroke, male mice developed a larger injury than females.^90^ Pro‐inflammatory agents such as caspases are the mediators of cell death in females, while the caspase‐independent Poly (ADP‐ribose) polymerase 1 (PARP‐1)‐mediated cell death and the nitric oxide pathway are more common in males poststroke (Figure 2).^6^ The pathways leading to the neuroinflammatory response poststroke also show differences between the two sexes. Examples of this are pathways involving peroxisome proliferator‐activated receptor alpha (PPAR‐α), high mobility group box 1 (HMGB1), and IL‐4 and IGF1 receptor signaling pathways (Figure 2).^6^ These pathways have known roles in microglial polarization and function, and it has been shown that PPAR‐α agonist treatment is only neuroprotective in males.^104^

The levels of cytokines and chemokines also differ between males and females after stroke, contributing to the variability in the inflammatory response between the two sexes. IL‐4 has been widely studied as a crucial anti‐inflammatory cytokine in stroke. Preclinical studies have shown that IL‐4 knockout mice (both males and females) had larger infarct volume and neurological scores after MCAO.^105^ However, when comparing the two sexes, the number of macrophages was lower in females and M2 phenotype microglia were higher in females, therefore showing reduced inflammation.^105^ The anti‐inflammatory marker Ym1 was also shown to increase neuroprotection in females when compared to males when microglia from female mice were transplanted in male mice (Figure 2).^90^ Furthermore, TLRs, which have been widely studied in the inflammatory responses to stroke, are responsible for the differences in poststroke dimorphism between the two sexes (Figure 2). Specifically, the injury‐induced TLR2 was completely reduced in galectin‐3 KO mice poststroke both in vitro and in vivo.^106^ Galectin‐3 ablation KO leads to a reduction in IGF‐1, which is a microglia‐regulated inflammatory protein, as presented in a mouse model of stroke.^106^ Taken together, these data suggest that the innate immune system is involved in the sexual dimorphism that exists between males and females after stroke. Another factor that may contribute to this is the role of sex hormones in combination with the inflammatory responses from microglia.

There is an increasing body of evidence that emphasizes the importance of estrogen as a neuroprotective agent after brain damage and ischemic injury. It has been determined that females are more protected from brain injury premenopause, while estrogen levels are higher. Postmenopause, estrogen levels decrease, and there is then an increased risk for stroke. Experimental models of stroke have found that estradiol has neuroprotective and anti‐inflammatory effects.^107^ However, clinical studies of hormone replacement have potential harmful effects in humans including breast and endometrial cancer as well as thrombosis.^6^ The reduction in estrogen levels postmenoupause explains the shift in the risk for stroke between males and females with age. Recent evidence suggests that chronic estrogen deficiency in postmenopausal women allows for increased activation of immune‐related genes^6^ and thus an exaggerated response to ischemic injury. Poststroke, chronically ovarian hormone‐deprived ovx, and ERα‐deficient mice had deregulated innate immune responses and microglial activation poststroke.^86^ These mice also had increased IL‐6 production, thus increasing the pro‐inflammatory response poststroke.^86^ Taken together, these data suggest that the lack of estrogen and estrogen receptors expression postmenopause can cause higher vulnerability to ischemic brain damage in aged females.

## 8 CONCLUSION

The sexual dimorphism that exists in stroke may have an influence on treatment strategies in the two sexes. There have been several promising preclinical therapeutic interventions for stroke; however, due to sex differences in poststroke responses they are not easily translatable to the clinic. Reasons for these include the differences in pro‐ and anti‐inflammatory protein expression between the two sexes, which can lead to sexually dimorphic cell death pathways.^6^ Tissue plasminogen activator (tPA) is the only clinically approved treatment for acute stroke. It has been determined that early responses with tPA are more effective in women.^108^ Research shows that males are more likely to receive tPA as a therapeutic option, whereas women may be excluded owing to outside risk factors (ie, hypertension).^109^ Furthermore, certain experimental anti‐inflammatory treatments, such as minocycline, which inhibits the PARP‐1 pathway, are more effective in males in both preclinical and clinical studies.^109^, ^110^ Lastly, IGF‐1, which can be microglial‐regulated, has shown promise as treatment in estrogen‐deficient middle‐aged females by reducing stroke‐induced damage and motor impairment in the aging brain.^34^ It is possible that the sexual dimorphism in microglial function may contribute to the differences that are seen in treatment effectiveness between the two sexes. Further research targeting microglial‐regulated inflammatory and sex hormone responses are necessary to further the development of pharmacological treatment poststroke.

## CONFLICT OF INTERESTS

Helen M. Bramlett and Dalton Dietrich are cofounders and managing members of InflamaCORE, LLC, a company dedicated to developing therapies and diagnostic tools focusing on the inflammasome.
