# Erratum

**DOI:** 10.1111/cns.13539

**Published:** 2020-12-14

In the following article,^1^ the name of the second author is listed incorrectly as Dalton W. Dietrich and should be listed as W. Dalton Dietrich.

The correct list of author names is listed below:

Nadine Kerr, W. Dalton Dietrich, Helen M. Bramlett, Ami P. Raval.
